# Design and validation of a multi-task, multi-context protocol for real-world gait simulation

**DOI:** 10.1186/s12984-022-01116-1

**Published:** 2022-12-16

**Authors:** Kirsty Scott, Tecla Bonci, Francesca Salis, Lisa Alcock, Ellen Buckley, Eran Gazit, Clint Hansen, Lars Schwickert, Kamiar Aminian, Stefano Bertuletti, Marco Caruso, Lorenzo Chiari, Basil Sharrack, Walter Maetzler, Clemens Becker, Jeffrey M. Hausdorff, Ioannis Vogiatzis, Philip Brown, Silvia Del Din, Björn Eskofier, Anisoara Paraschiv-Ionescu, Alison Keogh, Cameron Kirk, Felix Kluge, Encarna M. Micó-Amigo, Arne Mueller, Isabel Neatrour, Martijn Niessen, Luca Palmerini, Henrik Sillen, David Singleton, Martin Ullrich, Beatrix Vereijken, Marcel Froehlich, Gavin Brittain, Brian Caulfield, Sarah Koch, Anne-Elie Carsin, Judith Garcia-Aymerich, Arne Kuederle, Alison Yarnall, Lynn Rochester, Andrea Cereatti, Claudia Mazzà, for the Mobilise-D consortium

**Affiliations:** 1 Department of Mechanical Engineering and Insigneo Institute for in Silico Medicine, The University of Sheffield, Sheffield, UK; 2 Department of Mechanical Engineering, The University of Sheffield, Sheffield, UK; 3 Department of Biomedical Sciences, University of Sassari, Sassari, Italy; 4 Translational and Clinical Research Institute, Faculty of Medical Sciences, Newcastle University, Newcastle upon Tyne, UK; 5 Center for the Study of Movement, Cognition and Mobility, Neurological Institute, Tel Aviv Sourasky Medical Center, Tel Aviv, Israel; 6 Department of Neurology, University Medical Center Schleswig-Holstein Campus Kiel, Kiel, Germany; 7 Robert Bosch Gesellschaft für Medizinische Forschung, Stuttgart, Germany; 8 Laboratory of Movement Analysis and Measurement, Ecole Polytechnique Federale de Lausanne, Lausanne, Switzerland; 9 Department of Electronics and Telecommunications, Politecnico di Torino, Turin, Italy; 10 PolitoBIOMed Lab, Biomedical Engineering Lab, Politecnico di Torino, Turin, Italy; 11 Department of Electrical, Electronic and Information Engineering «Guglielmo Marconi», University of Bologna, Bologna, Italy; 12 Health Sciences and Technologies-Interdepartmental Center for Industrial Research (CIRI-SDV), University of Bologna, Bologna, Italy; 13 Department of Neuroscience and Sheffield NIHR Translational Neuroscience BRC, Sheffield Teaching Hospitals NHS Foundation Trust, Sheffield, UK; 14 Department of Sport, Exercise and Rehabilitation, Northumbria University Newcastle, Newcastle upon Tyne, UK; 15 Newcastle upon Tyne Hospitals NHS Foundation Trust, Newcastle upon Tyne, UK; 16 Machine Learning and Data Analytics Lab, Department of Artificial Intelligence in Biomedical Engineering, Friedrich-Alexander-Universität Erlangen-Nürnberg, Erlangen, Germany; 17 Insight Centre for Data Analytics, University College Dublin, Dublin, Ireland; 18 School of Public Health, Physiotherapy and Sports Science, University College Dublin, Dublin, Ireland; 19 Novartis Institutes of Biomedical Research, Novartis Pharma AG, Basel, Switzerland; 20 McRoberts BV, The Hague, The Netherlands; 21 Digital Health R&D, AstraZeneca, Sweden; 22 Department of Neuromedicine and Movement Science, Norwegian University of Science and Technology, Trondheim, Norway; 23 Grünenthal GmbH, Aachen, Germany; 24 Barcelona Institute for Global Health (ISGlobal), Barcelona, Spain; 25 Universitat Pompeu Fabra, Barcelona, Catalonia Spain; 26 CIBER Epidemiología y Salud Pública (CIBERESP), Madrid, Spain

**Keywords:** Digital mobility outcomes, Technical validation, Wearable sensors, Neurological diseases, Mobility monitoring

## Abstract

**Background:**

Measuring mobility in daily life entails dealing with confounding factors arising from multiple sources, including pathological characteristics, patient specific walking strategies, environment/context, and purpose of the task. The primary aim of this study is to propose and validate a protocol for simulating real-world gait accounting for all these factors within a single set of observations, while ensuring minimisation of participant burden and safety.

**Methods:**

The protocol included eight motor tasks at varying speed, incline/steps, surface, path shape, cognitive demand, and included postures that may abruptly alter the participants’ strategy of walking. It was deployed in a convenience sample of 108 participants recruited from six cohorts that included older healthy adults (HA) and participants with potentially altered mobility due to Parkinson’s disease (PD), multiple sclerosis (MS), proximal femoral fracture (PFF), chronic obstructive pulmonary disease (COPD) or congestive heart failure (CHF). A novelty introduced in the protocol was the tiered approach to increase difficulty both within the same task (e.g., by allowing use of aids or armrests) and across tasks.

**Results:**

The protocol proved to be safe and feasible (all participants could complete it and no adverse events were recorded) and the addition of the more complex tasks allowed a much greater spread in walking speeds to be achieved compared to standard straight walking trials. Furthermore, it allowed a representation of a variety of daily life relevant mobility aspects and can therefore be used for the validation of monitoring devices used in real life.

**Conclusions:**

The protocol allowed for measuring gait in a variety of pathological conditions suggests that it can also be used to detect changes in gait due to, for example, the onset or progression of a disease, or due to therapy.

*Trial registration:* ISRCTN—12246987.

**Supplementary Information:**

The online version contains supplementary material available at 10.1186/s12984-022-01116-1.

## Background

According to the World Health Organisation definition (2001) [1], mobility is “the activity of moving by changing body position or location or by transferring from one place to another, by carrying, moving or manipulating objects, by walking, running or climbing and by using various forms of transportation”. As with any other activity, mobility subsumes related qualifiers: performance and capacity. While performance qualifies what people do in their current environment, capacity aims to indicate the highest level of mobility that an individual may achieve in a given standardised environment.

Wearable devices such as inertial measurement units (IMUs) can be used to quantitatively assess both mobility qualifiers, with associated observations carried out in the form of short, structured tests (for example when instrumenting a six-minute walk test [2] or a Timed Up and Go test [3]) for mobility capacity or continuous unsupervised monitoring for mobility performance. In both cases, the signals from the IMUs are processed to extract specific features of mobility known as digital mobility outcomes (DMOs), such as the spatiotemporal parameters of gait. While algorithms to extract DMOs for capacity tests can be validated directly in a laboratory when equipped with a gold standard (e.g., 3D motion capture systems), the algorithm validation becomes much more complex when dealing with performance related DMOs. This is because measuring mobility in daily life entails dealing with confounding factors arising from multiple sources, including pathological characteristics, patient-specific walking strategies, environment/context, and purpose of the task. Accounting for all these factors within a single set of observations carried out within a limited laboratory space, while ensuring minimisation of participant burden and safety, is a very difficult and complex endeavour.

Previous work validating the estimation of DMOs has predominantly focused on standard straight walking assessments over a short distance [4–6] within a laboratory setting. Although this task is beneficial in gaining a measure under controlled conditions i.e. a measure of walking capacity, it does not consider any contextual factors or complexities in walking, which are necessary aspects to include when validating performance DMOs. In response, recent laboratory-based validations have proposed protocols that included assessments with more complex tasks such as stair negotiation [7], incline walking [9], inclusion of single and dual tasks [10], variation in walking speed (WS) imposed via a treadmill [11, 12] or overground [13], as well as variation of walkway surfaces [14] and curvilinear paths [8, 15]. The fact that these studies mostly attempt to validate specific DMOs related to the task (e.g., validation of a cadence algorithm during stair ascent [7] or detecting variation of temporal parameters on different walking surfaces [14]) limits the generalisability of their results and more complex scenarios mimicking the real-world have hence been proposed. These approaches attempt to simulate daily life environments for the purpose of validating continuous monitoring devices and usually entail the participant moving freely within a lab setting while completing a series of goal-oriented tasks designed to mimic the postures and movements expected to be seen in the real-world. In accordance with this concept, Bourke et al. [16] identified a subset of tasks from the Compendium of Physical Activity [17], to include as many variations as possible of real-world walking and associated postural transitions. This led to a protocol that had approximately 30 min of activity data for each participant, when deployed in a group of healthy older adults, and included 134 tasks with multiple repetitions of different transitions and straight walking at varying speeds. With such a comprehensive set of tasks however, this protocol is unsuitable for assessing individuals with reduced levels of mobility. Subsequently, protocols with a more refined list of tasks have been proposed by other authors to reduce repetitions [18] or accommodate the inclusion of patients with Parkinson’s Disease [19], but with these home-like assessments lasting 90–180 min the duration is still such that only a simple gold standard (such as 2D videos) could be used, limiting the validation of the monitoring device to activity recognition or basic gait parameters like step detection. Warmerdam et al. [20] recently proposed a much shorter, and hence more feasible home-like assessment that was situated in the volume of a 3D motion capture system, but as the main aim of this set of tasks was to observe changes in balance and postural control the translation of this assessment when aiming to mimic real-world walking would not be effective. Overall, a comprehensive protocol that could effectively mimic a variety of complex walking patterns within a lab setting and be safely administered to participants with different levels of mobility has yet to be identified.

When attempting to move the validation of estimating DMOs to a real-world context, significant hurdles are associated with the feasibility of deploying a gold standard and ensuring a meaningful amount of data are collected. Although several technological solutions have recently been proposed in the literature, including GO-Pro body-worn cameras [16, 21], footswitches or pressure insoles [22], foot or ankle-mounted IMUs [23], and multi-sensor systems that integrate IMUs and infrared distance sensors [24], the complexity of processing data from some of these systems often limits the duration of the observation. With no clear information in the literature about how long these real-world observations should last and which tasks must be included while still preserving an acceptable level regarding the burden and cognitive demand asked of the participant, an assessment of the duration required to ensure a meaningful amount of data are recorded while keeping in mind the above considerations is necessary. For example, if the attention is focused on assessing gait performance, data from an adequate number of walking bouts (WBs) should be collected and these should be recorded within complex and representative contexts, such as inclined walking, stair negotiation and indoor and outdoor settings.

Within this framework, the primary aim of this study is to validate a multi-task and multi-context protocol for simulating real-world gait. The protocol includes a laboratory-based assessment and a 2.5 h unsupervised data collection in the participants’ habitual environment. Validation of this protocol will focus on proving that the chosen series of complex activities in the laboratory-based assessment: (a) are suitable for the evaluation of a variety of gait patterns, including healthy gait and impaired gait associated with neurodegeneration, a proximal femoral fracture, chronic pulmonary disease or congestive heart failure; (b) include at least one WB, defined as a minimum of two consecutive strides of both feet [25]; (c) induce a large variation in gait strategies, resulting in a broad range of WSs captured; and (d) avoid redundancy in the tasks to minimise burden to the participant. In addition, a secondary aim of the validation is to determine whether 2.5 h of unsupervised monitoring in the participants’ habitual environment is a long enough observation to collect a set of data that is extensive and reliable for assessing the validity of gait related DMOs in a real-world context. We expect that these results will establish a common ground for the technical validity of wearable devices aimed at estimating gait related DMOs in real-world settings.

## Methods

### Data collection

As part of an observational study (Mobilise-D [26]), a convenience sample of 108 participants were recruited from six cohort groups that included older healthy adults (HA) and participants with potentially altered mobility due to Parkinson’s disease (PD), multiple sclerosis (MS), proximal femoral fracture (PFF), chronic obstructive pulmonary disease (COPD) or congestive heart failure (CHF). These cohorts were chosen as presenting a variety of gait and mobility features. Besides the cohort specific inclusion and exclusion criteria (see Additional file 1: Table S1 and [27] for more details), all participants were: (1) able to give informed consent, (2) willing to wear the sensors setup and participate in the different data collections of the study, (3) scored >15 in the Montreal Cognitive Assessment (MoCA), (4) were able to walk at least 4m, (5) had no comorbidities impacting mobility or compliance. Data were collected across five gait laboratories after receiving written informed consent (Ethics approvals: The Newcastle upon Tyne Hospitals NHS Foundation Trust and Sheffield Teaching Hospitals NHS Foundation Trust: London—Bloomsbury Research Ethics committee, 19/LO/1507; Tel Aviv Sourasky Medical Center: the Helsinki Committee, 0551-19TLV; Robert Bosch Foundation for Medical Research: medical faculty of the University of Tübingen, 647/2019BO2; University of Kiel: medical faculty of Kiel University, D540/19). Participant demographics were collected, and patient characterisation was completed based on clinical assessments specific to each cohort [27] (Table 1).

**Table 1:** Summary of participant demographics and clinical characteristics (mean ± standard deviation)

Characterisation of groups						
	HA (n = 20)	PD (n = 20)	MS (n = 20)	PFF (n = 19)	COPD (n = 17)	CHF (n = 12)
*Generic characteristics*						
Sex (Male/Female)	11/9	16/4	11/9	8/11	9/8	8/4
Age (years)	71.7 ± 5.8	69.8 ± 7.2	48.7 ± 9.7	80.0 ± 8.5	69.4 ± 9.1	69.1 ± 11.7
Height (m)	1.66 ± 0.10	1.73 ± 0.07	1.71 ± 0.13	1.69 ± 0.08	1.69 ± 0.07	1.74 ± 0.10
Weight (kg)	75.1 ± 11.8	78.2 ± 14.4	84.0 ± 22.9	68.4 ± 16.0	73.7 ± 14.2	84.5 ± 16.8
*Cognition*						
MoCA^†^	27.7 ± 2.6	24.6 ± 4.0	26.7 ± 3.1	24.1 ± 4.2	24.6 ± 3.4	27.1 ± 2.9
*No. of fallers*						
Had a fall in last 12 months	3	10	11	19	1	3
*Pain—VAS Score (0, no pain – 100 worst)*						
General	11.1 ± 18.6	20.5 ± 26.4	23.4 ± 24.7	13.2 ± 16.9	14.1 ± 16.1	16.8 ± 28.1
When walking	8.1 ± 13.9	21.8 ± 27.2	26.4 ± 32.5	25.5 ± 27.4	13 ± 14.4	17.8 ± 30.1
*No. of walking aid users*						
General use	1	6	5	13	1	4
Laboratory protocol	0	1	3	6	0	4
*Cohort-specific outcomes*						
LLFDI^†^	73.53 ± 14.22	60.26 ± 12.51	57.34 ± 10.66	52.59 ± 16.61	59.07 ± 7.96	67.29 ± 21.35
UPDRS III^†^	–	28.4 ± 13.6	–	–	–	–
H&Y Score^†^	–	I n = 4, II n = 11, III n = 5	–	–	–	–
EDSS^†^	–	–	3.5 ± 1.7	–	–	–
SPPB^†^	–	–	–	6.2 ± 3.9	–	–
CAT score^†^ (0, best–40, worst)	–	–	–	–	16.6 ± 8.9	–
FEV_1_^†^ (L)	–	–	–	–	1.6 ± 0.6	–
FVC^†^ (L)	–	–	–	–	2.9 ± 0.7	–
6MWT^†^ distance (m)	–	–	–	–	357.6 ± 88.5	370.7 ± 115.6
KCCQ^†^	–	–	–	–	–	80.5 ± 20.2

^†^Acronyms used in table for: Montreal Cognitive Assessment (MoCA), Late-Life Functional and Disability Instrument (LLFDI), Unified Parkinson’s Disease Rating Scale (UPDRS), Hoehn and Yahr Score (H&Y), Expanded Disability Status Scale (EDSS), Short Physical Performance Battery (SPPB), COPD Assessment Test (CAT), Forced expiratory volume (FEV1), Forced vital capacity (FVC) six-minute walk test (6MWT) and Kansas City Cardiomyopathy Questionnaire (KCCQ)

### Experimental protocol

#### Laboratory-based assessment

Based on previous literature [16, 17], five key elements associated with walking in real-world scenarios were identified as necessary to vary in the multi-task protocol: speed, incline/steps, surface, path shape, and cognitive demand. In addition, specific motor tasks and postures that may abruptly alter the participants strategy of walking were included to further broaden the simulation of typical real-world transitions (e.g., walk-to-sit).

Besides including a large variation in walking paths and transitions, a critical target for the analysis was that the desired DMOs could be calculated for each WB. The focus was specifically placed on WS as a summary measure of these walking variations. In the context of this study, a WB was defined as a period of walking that included at least two consecutive strides for each leg [25]. When considering normative values for stride length at approximately 1.1–1.5 m [28], this definition safely translates into a minimal travelled distance of 3.5 m for each period of walking. For the laboratory-based assessment, given the limitations of the 3D motion capture systems within the five gait laboratories involved in the study, the protocol capture area was designed to be smaller than 5 m×4 m.

**Fig. 1:**
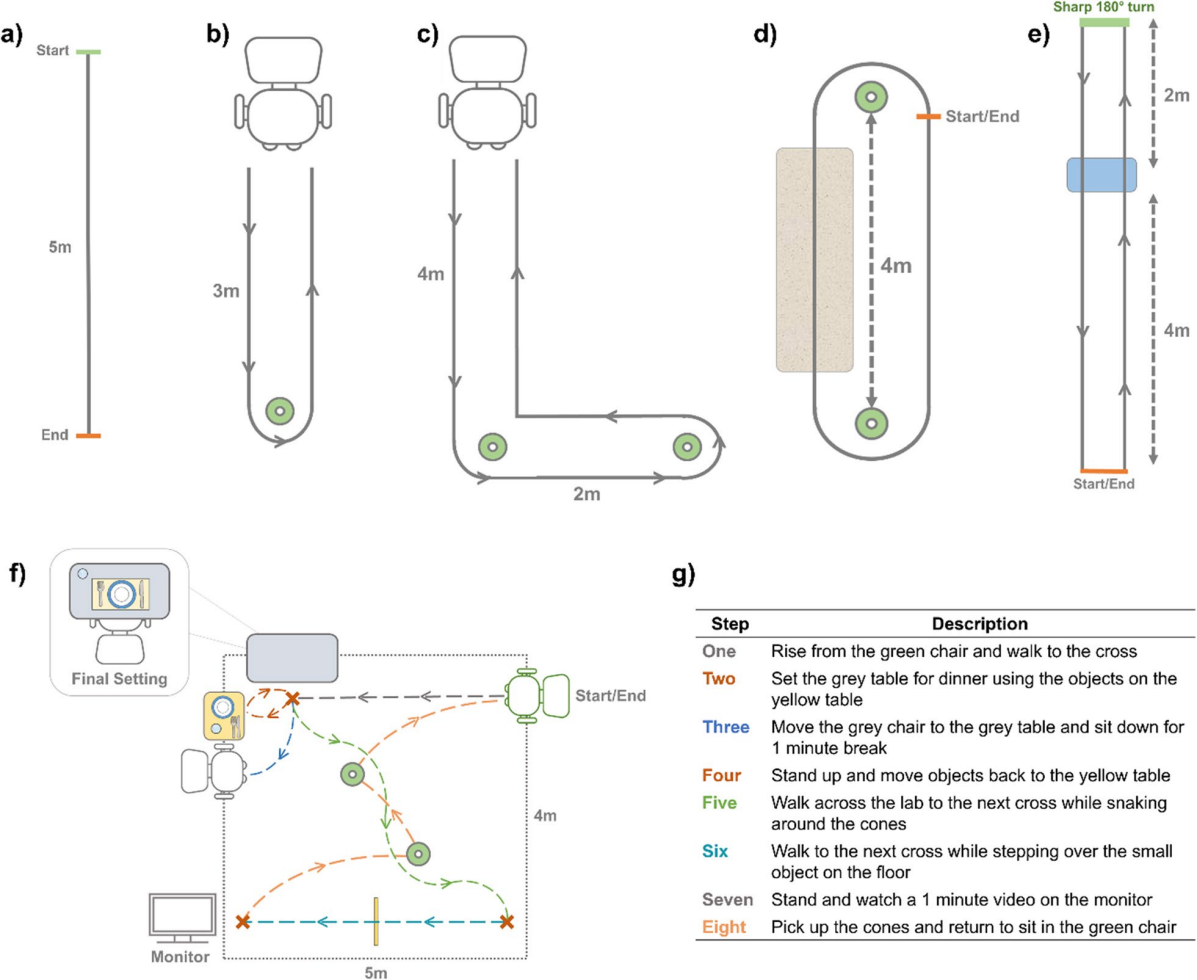
Schematics of the seven structured tasks: **a** straight walking, **b** TUG, **c** L-test, **d** Surface test and **e** Hallway test completed in the laboratory-based assessment, as well as a schematic of the simulated daily activities (**f**) and description of the eight steps performed during this task (**g**)

In light of the above considerations, the designed protocol included seven structured tasks with each task varying in at least one of the identified elements of a walking path that are subject to change in real-world scenarios, as well as variation in postural transitions at the start and end of each task. In addition, a task that focused on simulating daily activities was designed, that resulted in the most complex combinations of walking paths and transitions [27]. The eight tasks were performed in the order presented below and only performed once for each participant, except for the straight walking trials that were performed twice. All tasks were described and demonstrated to the participant by the researcher prior to completing the task, allowing the participant the opportunity to determine if they felt comfortable and safe performing the task. In addition, adjustments (e.g., whether the participant wished to use their walking aid and/or modulation to the task difficulty, as described below) was determined by the participant and researcher at this stage:

- Straight Walking: Participants were asked to walk a predefined path of 5 m from a standing start to a standing end. This trial was performed twice at three self-selected speeds: comfortable, slow, and fast (Fig. 1a).
- Timed Up and Go (TUG): At a comfortable speed, participants were asked to rise from the chair and walk 3 m to the cone, make a 180° left hand turn around the cone, walk back to chair and sit down (Fig. 1b).
- L-Test: At a comfortable speed, participants were asked to rise from the chair and walk 4 m to the first cone, make a 90° turn to the left around the cone and walk straight to the second cone, turn 180° to the left around the cone and walk straight before making a final 90° turn to the right of the first cone and walking back to sit in the chair (Fig. 1c).
- Surface Test: Participants were asked to walk at a comfortable speed around the circuit by turning around the cones and stepping over the carpeted mat completing the circuit twice. Participants started and stopped the tasks in a standing position (Fig. 1d).
- Hallway Test: From a standing start, participants were asked to walk at a comfortable speed along the predefined walkway stepping up and down from the step. At the end of the walkway participants completed a sharp 180° turn and walked back along the walkway (again stepping up and down the step) before coming to a stop at the end of the walkway (Fig. 1e).
- Simulated Daily Activities: Participants were asked to start sitting in the green chair (Fig. 1f) and complete a series of tasks defined in Fig. 1g. while moving around the room. The tasks were split into separate steps, with the next set of instructions only given to the participant after the previous step had been completed. All steps for this task were completed at the preferred walking speed of the participant.

This multi-task design allowed for a tiered approach to the data collection, with tasks set in ascending order of complexity (based on consensus of the authors) to allow participants to ask to stop the data collection at any point that the protocol became too burdensome. The grade of difficulty within each task was also modulated to accommodate the use of walking aids, arm rests for the TUG, L-test and Simulated Daily Activities, handrails for the step in the Hallway test and by removing obstacles from the Simulated Daily Activities path to facilitate foot clearance. This tiered approach was deemed appropriate to account for the wide variation in physical health and level of mobility across the sample populations to ensure that a meaningful amount of data could be collected for all participants while safeguarding the participants’ safety and well-being.

**Fig. 2:**
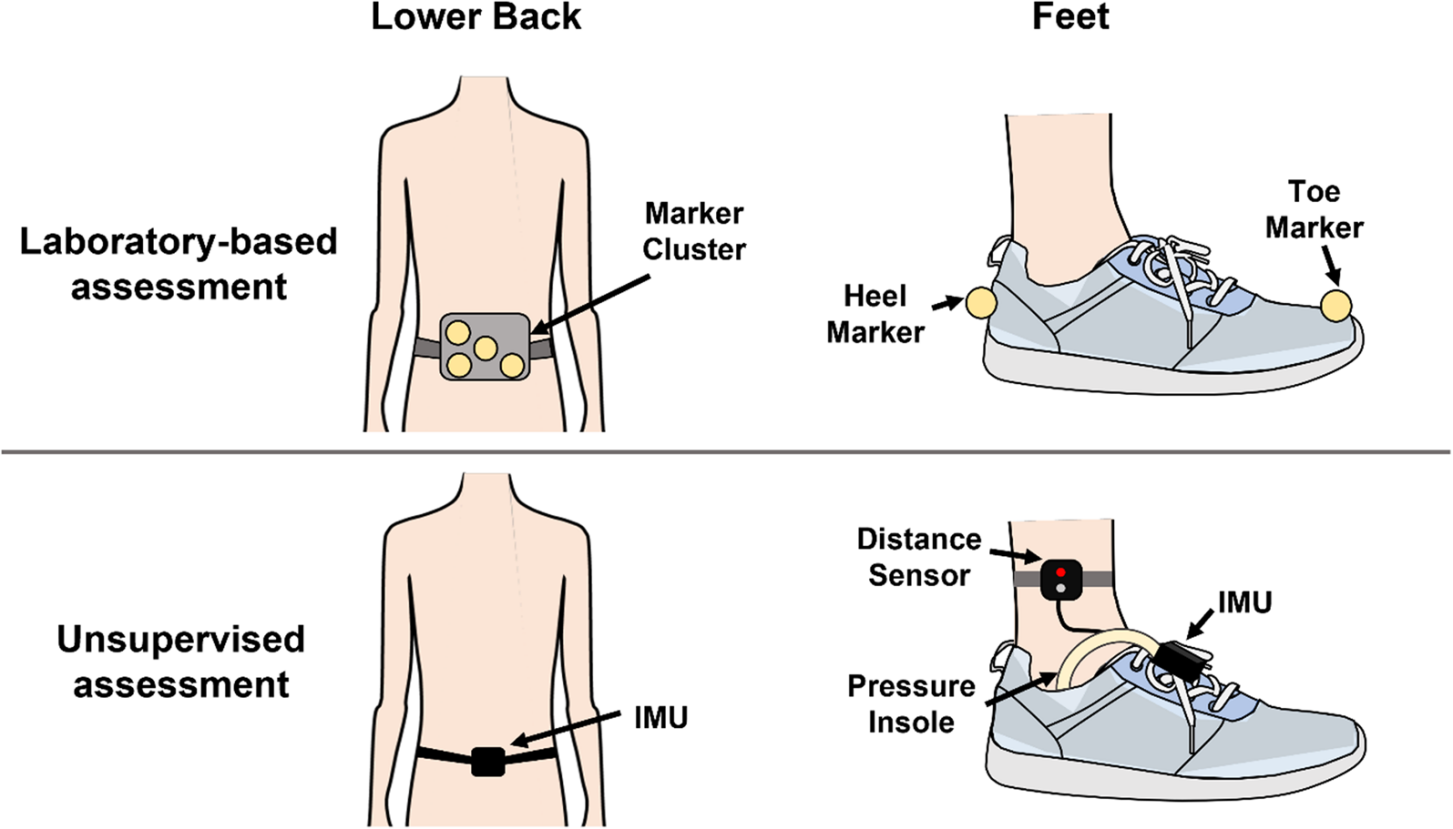
Illustration of the participant setup in the laboratory assessment (marker configuration used that included a marker cluster on the lower back and markers placed on the dorsal aspect of second metatarsal head and heel of each foot) and the multi-sensor system worn during the unsupervised assessment, that consisted of IMUs on the lower back and both feet, as well as 16-point pressure insole in each shoe and infrared time-of-flight distance on both legs (at a comfortable height above the medial malleolus)

During all the above tasks, a 3D motion capture system was used to record the trajectories of two markers located on each foot (Heel and Toe) and a four-marker cluster on the lower back (Fig. 2). The temporal and spatial parameters needed to compute WS were calculated using the marker trajectories for each detected WB, as described in Bonci et al., [29].

#### Unsupervised assessment

In addition to the laboratory-based assessment, all participants underwent an unsupervised data collection in their habitual environment. During this session participants were asked to go about their typical routine and consider incorporating some specific tasks to ensure the presence of some variability/crucial elements in the data collection: outdoor walking; walking an inclined or declined path; moving from one room to another; walking up and down stairs. WS was recorded using a multi-sensor wearable system (INertial module with DIstance sensors and Pressure insoles, INDIP) integrating multiple IMUs, 16-points pressure insoles and infrared time-of-flight distance sensors (Fig. 2) [30, 31]. Gait events were detected by the pressure insoles and foot IMUs of the INDIP system and combined, with priority given to the events detected by the pressure insoles if detected by both methods [30], which allowed the calculation of the temporal parameters for each detected WB. Spatial parameters were then calculated from the foot IMUs data based on the direct and reverse integration approach described in [31]. The duration of this session was set to 2.5 h to minimise participant burden and safely operate within the limits imposed by the battery integrated in the INDIP system.

When compared to the marker trajectory method used in the laboratory-based assessment, the INDIP system estimated walking speed with a median absolute error of 0.02 m/s [30, 31]. It was hence deemed appropriate to use bins of 0.1 m/s for comparing the data obtained from the different systems used in the laboratory and in the real-world observation.

### Data analysis

Once WBs were identified for each task and participant, the relevant WS was calculated as the average stride speed over the considered WB.

$$WS= \frac{\sum_{k=1}^{{n}_{strides}}{StrideSpeed}_{k}}{{n}_{strides}}$$

where *n*_*strides*_ is the number of strides identified in a given WB and ${StrideSpeed}_{k}$ is the walking speed of stride *k* in the WB, defined as:

$${StrideSpeed}_{k} [m/s]= \frac{{StrideLength}_{k} [m]}{{StrideDuration}_{k} [s]}$$

When more than one WB was detected within a task (as expected with the Simulated Daily Activities), the WS values for all WBs belonging to that task were included. The frequency and distribution of the WSs recorded in the lab were then computed for each cohort.

To establish whether the 2.5 h of unsupervised recording was sufficient to reach an adequate sample size from a statistical point of view, a preliminary statistical power analysis was conducted to define the minimum number of WBs for each cohort required to validate the estimate of WS. In order to obtain a confidence interval smaller than 0.1 when comparing two different instruments with an α = 0.05 and a power ß = 0.9, an analysis was performed in Stata 16.1 (Stata Corp LP; College Station, Texas, USA; command line: sampicc 0.7 2, alpha (0.05) power (0.9) w (0.1) ci), which showed that the minimum number of WBs needed in each cohort for an Intraclass Correlation Coefficient ICC ≥ 0.7 would be 401.

Finally, to determine whether the laboratory-based protocol could mimic the same WS range as recorded in the 2.5h assessment, the minimum and maximum walking speeds were extracted for all WBs recorded both in the supervised and unsupervised testing. The bias and limits of agreement of these two variables were then calculated and used to create Bland Altman plots for each cohort.

## Results

### Protocol safety and feasibility

The protocol proved to be safe and feasible. No adverse events were recorded in either stage of the data collection, despite the fact that it was administered to patients with severe mobility impairments and during the COVID-19 pandemic.

**Fig. 3:**
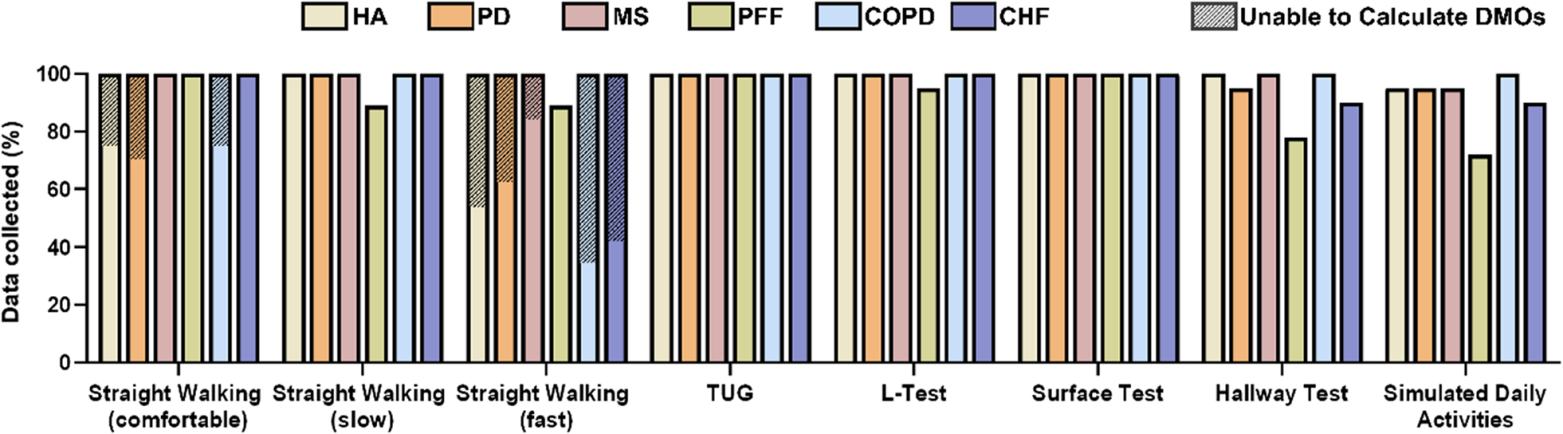
Data successfully collected for each laboratory-based task in percentage of planned. Striped portions of the bar are from trials in which data was collected but not enough strides were collected to identify a WB and calculate WS

Regarding the laboratory based assessment, from the 108 participants included, 100% managed to complete the straight walking at a comfortable speed, the TUG, and the surface test; > 95% completed the slow and fast walking tasks and the L-test, and > 85% also completed the Hallway test and the Simulated Daily Activities (see details in Fig. 3). Participants that were not able to complete some of tasks were always from a patient cohort and were generally reported to be more severely affected by their individual disease based on the cohort specific outcomes.

The design of the protocol allowed calculation of WS and other DMOs in the vast majority of the recorded tasks, with the exceptions being mostly in the fast speed walking (Fig. 3), where the limited available space did not allow the recording of enough strides to satisfy the WB definition criteria. Those participants for which DMOs were not calculated were primarily within the healthy older adult population or participants who exhibited milder disease severity as indicated by the lower clinical scores.

**Fig. 4:**
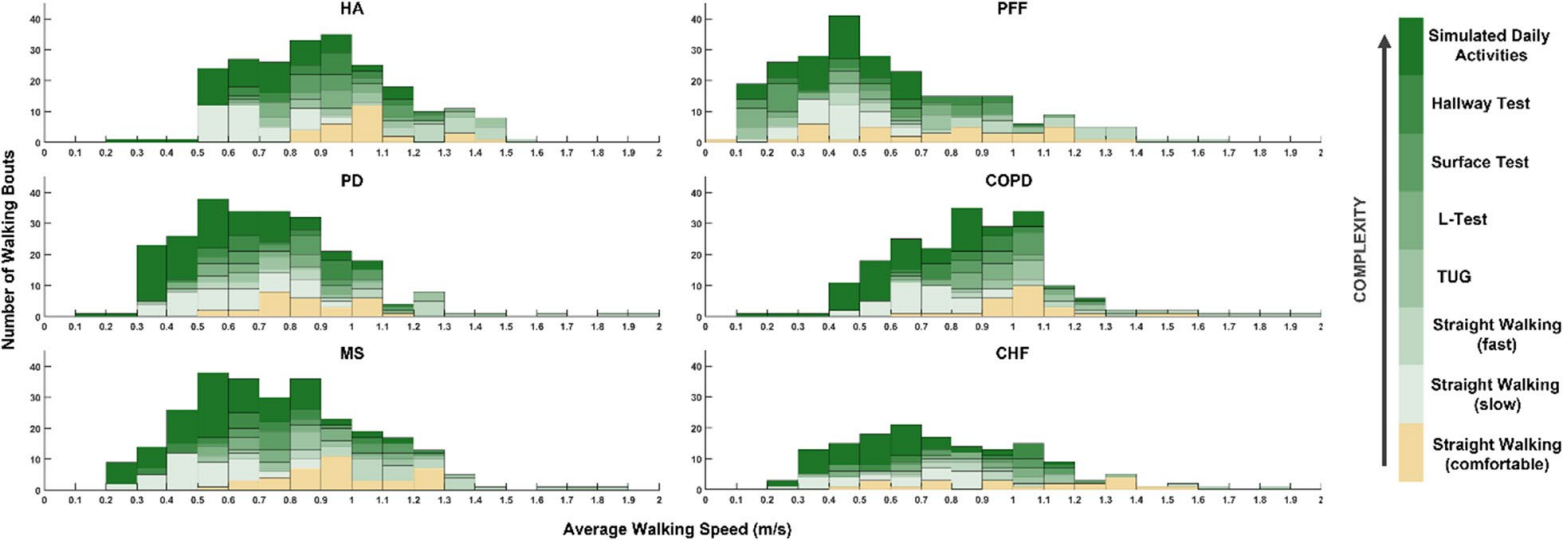
Stacked bars representing the distribution and frequency of the average walking speeds collected for the laboratory-based protocol. The colouring represents the contribution from individual tasks to the overall number of WB recorded at each speed, with orange bars highlighting the Straight Walking tasks at a comfortable speed. The green bars are the other tasks, with the darker colour related to the higher complexity of the task. Data used to generate the graphs is available in Additional file 2

As per the third protocol design objective of inducing a variety of gait patterns, a large range of WSs were captured for each cohort (Fig. 4). In particular, the addition of the more complex tasks allowed a much greater spread in WSs to be achieved compared to the standard straight walking trials. The distribution of the WSs recorded for the PFF patients was skewed by the lowest speeds, but overall, the protocol allowed the same range of speeds to be observed for the other groups.

### Duration of real-world observation

**Fig. 5:**
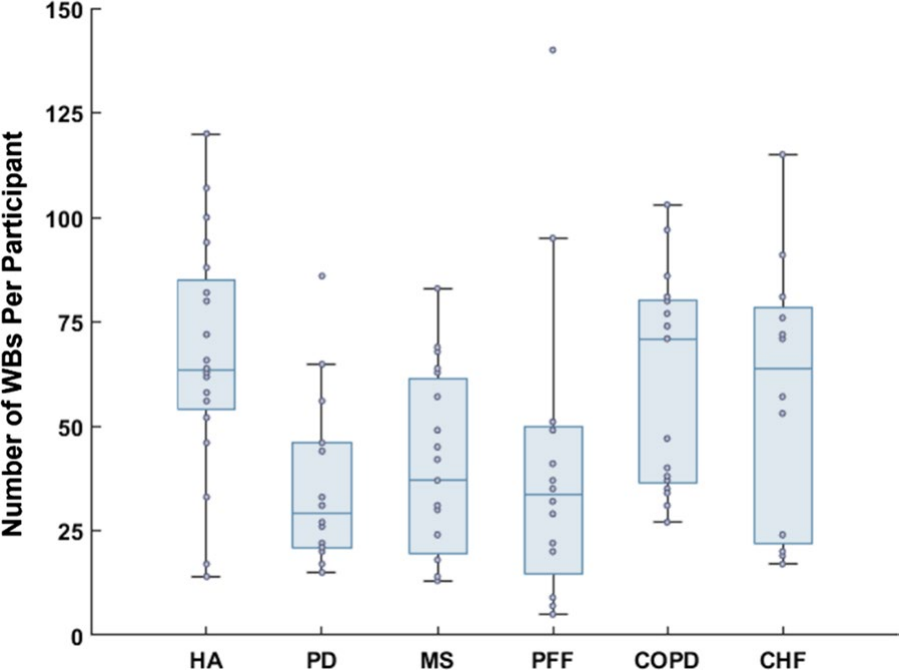
Box plot of the total number of WBs recorded for each cohort, with data points for each participant, during the 2.5h unsupervised data collection in the participants’ habitual environment

From the 108 participants recruited in this data collection, data from 102 were included in the analysis of the real-world observation (HA = 20, PD = 18, MS = 19, PFF = 16, COPD = 17 and CHF = 12). The reduced number was the result of technical complications with the synchronisation of the multiple systems used or experimental error during the observation. Figure 5 shows the total number of walking bouts recorded for each participant and cohort. In total, these were 1330 for the HA, 678 for the PD, 771 for the MS, 628 for the PFF, 1035 for the COPD and 696 for the CHF cohort.

**Fig. 6:**
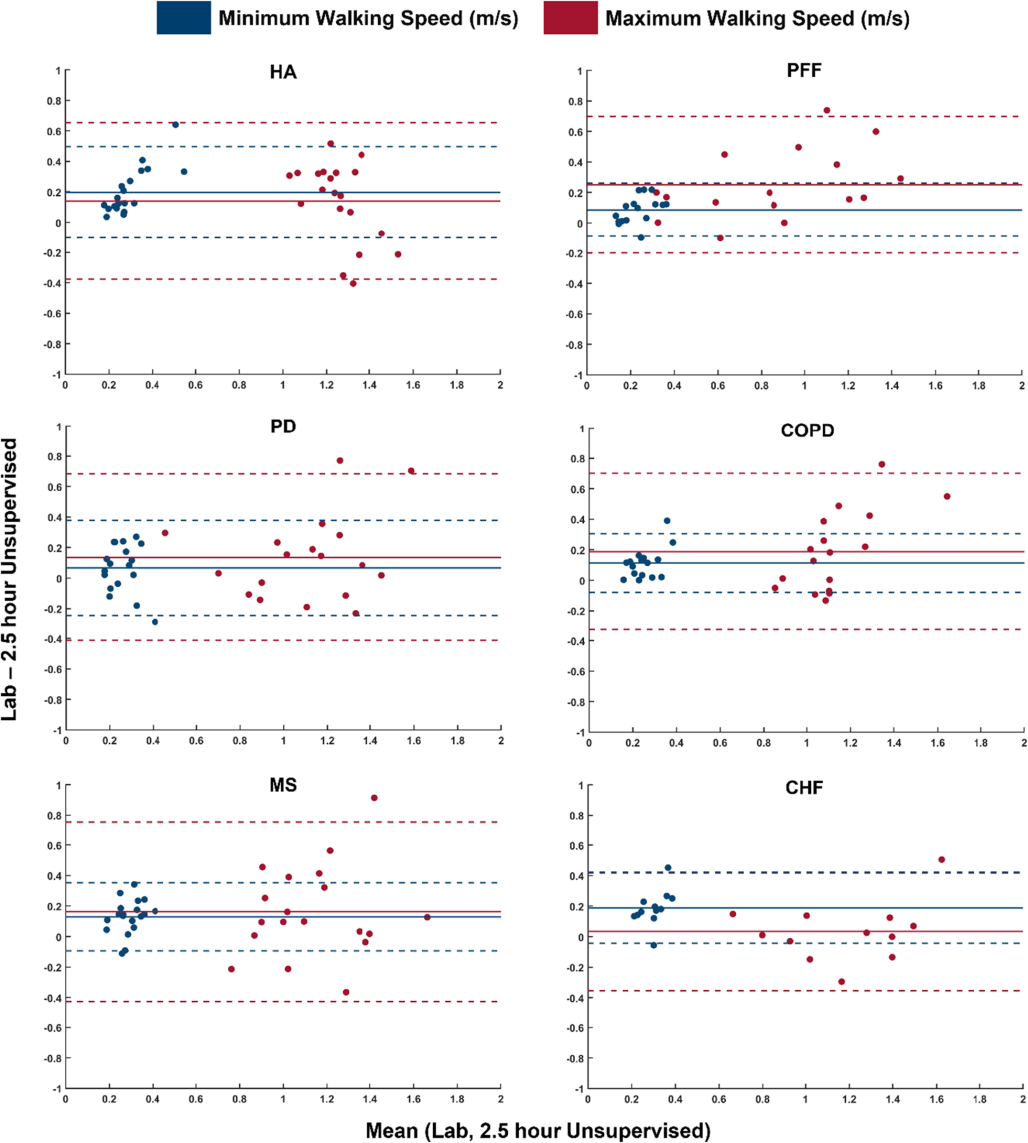
Bland–Altman plots of the minimum (blue) and maximum (red) walking speed recorded in the laboratory-based protocol compared to 2.5 h of unsupervised monitoring in a habitual environment. The solid horizontal lines (−) indicate the mean bias and dashed horizontal lines (–) the upper and lower limits of agreement (LOA). Data used to generate the graphs is available as Additional file 4

At participant level, the range of speeds observed in the lab was smaller than the one observed in real-world (see Additional file 3: Table S2 for more details). Figure 6 shows the LOA and bias of the minimum (blue) and maximum (red) walking speeds collected in the laboratory versus the values recorded during the 2.5 h unsupervised assessment for each participant. The minimum and maximum walking speeds observed in the real-world were generally slower than those recorded in the laboratory. However, these differences varied between cohorts, with the largest mean bias observed across all groups being 0.3 m/s for the maximum walking speed of PFF. In all groups, the limits of agreement for the maximum speeds were bigger than those observed for the minimum speeds.

## Discussion

This paper validated a protocol designed for simulating real-world gait in a laboratory setting. The reported results show that the proposed protocol was successfully designed and met its objectives. All participants were able to complete the majority (five out of seven) of the laboratory-based tasks, with the data collection typically lasting about 45 min. The selected tasks allowed for a broad range of WB speeds to be recorded in all groups, irrespective of their gait impairments. Furthermore, the 2.5 h unsupervised observation in the participants’ habitual environment allowed recording a number of WBs that was higher than the 401 threshold needed to ensure reliability and validity of a device for real-world gait monitoring in a given cohort.

A novelty introduced in the protocol was the tiered approach to increase difficulty with each new task while allowing for some adaptations to task setup to ensure inclusivity and safety of all participants (e.g., by allowing use of walking aids or armrests), with the Hallway test and Simulated Daily Activities being the most challenging. This ensured that the more severely disabled participants could opt out of tasks if they felt tired, unsafe, or too challenged, while also allowing for a variety of data to be collected. This proved to be a necessary and effective approach: the participants unable to complete some of the more complex tasks were all within the groups from a patient group and were rather severely affected by their individual disease based on the cohort specific outcomes. Notably, previous studies adopting complex protocols similarly succeeded in collecting a variety of gait data only included a few groups of participants, primarily older healthy adults [13–15] and individuals with PD [14, 15]. The successful inclusion of participants presenting with different types of pathological gait, is a clear indication of the protocol’s suitability to be safely and effectively deployed also in vulnerable groups.

The results showed that increasing task complexity allowed broad ranges of WSs to be captured for all participants, in comparison to only including tasks at natural speed, as is commonly the case in validation studies [2–4]. This was particularly evident for the HA and COPD cohort, where most participants walked at very similar speeds (HA 0.8–1.2 m/s and COPD 0.9–1.2 m/s) in the straight walking tests. Interestingly, asking the participants to perform the most complex tasks, namely the Hallway test and the Simulated Daily Activities, proved more effective at inducing slower walking speeds than simply asking participants to walk slowly. This can be explained by the increase in motor and cognitive demand associated with the tasks, in which the majority of walking periods included a change in walking environment (i.e., obstacles to avoid or stepover on the ground) or some form of dual task (i.e., carrying an object and walking), which are known to induce a speed reduction both in healthy and pathological groups [32] in more complex or noisy environments [33].

When leaving the laboratory and moving to the unsupervised observations in the habitual environment, the open question was around the effectiveness of the protocol in providing enough WBs to ensure a robust validation of the WS estimates. Setting the duration to 2.5 h and requesting that participants to consider incorporating some specific tasks within their unsupervised activities proved to be effective in all groups as reflected by the number of WBs recorded at group level. This was despite the different group sizes and the range of mobility disability exhibited. In general, as expected, the participants with the higher levels of severity in regards to their condition were those for whom the lower number of WBs were recorded.

When comparing data from the laboratory and the real-world sessions, the minimum and maximum WS results highlighted an overall bias with higher WSs recorded in the laboratory. Although this may not be surprising, the mean bias for both maximum and minimum walking speeds for all, aside from the maximum walking speed for PFF, fell below 0.2 m/s. Though this bias can be considered low compared to the larger biases reported in the literature for healthy adults, Parkinson’s disease, and multiple sclerosis [34, 35] it is likely explained by the fact that previous studies have included a much longer period of observation (e.g., 7 days data). While this aspect does not affect the usefulness of the 2.5 h observation for the purposes of gathering enough validation data, it is certainly an indicator of the likelihood that longer periods of continuous monitoring are needed when interested in assessing an individual’s mobility performance. Further studies would be required to further investigate this aspect and establish the link between capacity and performance assessments.

The results come with their limitations. The unbalanced size of the observed groups, which resulted from recruiting extremely vulnerable groups during the COVID-19 pandemic did not hinder our ability to achieve the study’s primary aims, but it certainly prevented further investigation on the differences between the groups and on the effect of specific disease severity. From a more practical perspective, another limitation sits in the fact that in the context of this multicentric study the walkway length (5 m) was dictated by the 3D motion capture system volume. The confinements to a small space did not allow the recording of more than four consecutive strides in the same direction: this is even more evident during the straight walks, in particular for participants with higher walking speeds and longer stride length, rather than during complex tasks including curvilinear portions, which cover longer distances in the capture volume. We would certainly recommend future studies consider extending this walkway length.

## Conclusions

This study presents an innovative multi-task gait assessment protocol beyond straight walking. It allows a relatively realistic representation of daily life relevant mobility aspects and can therefore be used for the validation of monitoring devices used in real life. Of particular note is the suitability of the protocol for measuring gait in conditions typically associated with pathological gait. This suggests that it can also be used to detect changes in gait due to, for example, the onset or progression of a disease, or due to therapy. Ultimately, this protocol opens up the option of capturing entirely new aspects of gait in real life, such as balance control in response to obstacles, directional behaviour, and cognitive aspects of mobility.

## Supplementary Information

**Additional file 1: Table S1. **Inclusion and exclusion criteria adopted for the different disease cohorts.

**Additional file 2.** Walking speed data from the laboratory-based assessment.

**Additional file 3: Table S2. **Descriptive statistics of the walking speed captured in the laboratory-based assessment and 2.5 hour of unsupervised monitoring in the participants habitual environment for each cohort.

**Additional file 4.** Maximum and minimum walking speed data from the laboratory-based assessment and 2.5 hour unsupervised assessment.

## References

[1] World Health Organisation (2001). The International Classification of Functioning, Disability and Health (ICF).

[2] ATS Committee on Proficiency Standards for Clinical Pulmonary Function Laboratories. ATS statement: guidelines for the six-minute walk test. Am J Respir Crit Care Med. 166(1): 111–7. 10.1164/ajrccm.166.1.at1102 10.1164/ajrccm.166.1.at1102 12091180

[3] Podsiadlo D, Richardson S (1991). The timed “Up & Go”: a test of basic functional mobility for frail elderly persons. J Am Geriatr Soc.

[4] Han YC, Wong KI, Murray I (2019). Stride length estimation based on a single Shank's gyroscope. IEEE Sens Lett.

[5] Micó-Amigo ME, Kingma I, Ainsworth E, Walgaard S, Niessen M, van Lummel RC, van Dieën JH (2016). A novel accelerometry-based algorithm for the detection of step durations over short episodes of gait in healthy elderly. J Neuroeng Rehabil.

[6] Aich S, Pradhan PM, Park J, Sethi N, Vathsa VSS, Kim H-C (2018). A validation study of freezing of gait (FoG) detection and machine-learning-based FoG prediction using estimated gait characteristics with a wearable accelerometer. Sensors.

[7] Zhou H, Ji N, Samuel O, Cao Y, Zhao Z, Chen S, Li G (2016). Towards real-time detection of gait events on different terrains using time-frequency analysis and peak heuristics algorithm. Sensors.

[8] Mariani B, Jiménez MC, Vingerhoets FJG, Aminian K (2013). On-shoe wearable sensors for gait and turning assessment of patients with Parkinson's disease. IEEE Trans Biomed Eng.

[9] Martinez-Hernandez U, Dehghani-Sanij AA (2018). Adaptive Bayesian inference system for recognition of walking activities and prediction of gait events using wearable sensors. Neural Netw.

[10] Sejdić E, Lowry KA, Bellanca J, Perera S, Redfern MS, Brach JS (2015). Extraction of stride events from gait accelerometry during treadmill walking. IEEE J Transl Eng Health Med.

[11] Dijkstra B, Zijlstra W, Scherder E, Kamsma Y (2008). Detection of walking periods and number of steps in older adults and patients with Parkinson's disease: accuracy of a pedometer and an accelerometry-based method. Age Ageing.

[12] McGinnis RS, Mahadevan N, Moon Y, Seagers K, Sheth N, Wright JA (2017). A machine learning approach for gait speed estimation using skin-mounted wearable sensors: from healthy controls to individuals with multiple sclerosis. PLoS ONE.

[13] Lee JK, Park EJ (2011). Quasi real-time gait event detection using shank-attached gyroscopes. Med Biol Eng Compu.

[14] Allet L, Armand S, de Bie RA, Golay A, Monnin D, Aminian K, de Bruin ED (2008). Reliability of diabetic patients’ gait parameters in a challenging environment. Gait Posture.

[15] O’Brien MK, Hidalgo-Araya MD, Mummidisetty CK, Vallery H, Ghaffari R, Rogers JA (2019). Augmenting clinical outcome measures of gait and balance with a single inertial sensor in age-ranged healthy adults. Sensors.

[16] Bourke AK, Ihlen EAF, Bergquist R, Wik PB, Vereijken B, Helbostad JL (2017). A physical activity reference data-set recorded from older adults using body-worn inertial sensors and video technology—the ADAPT Study Data-Set. Sensors.

[17] Ainsworth BE, Haskell WL, Leon AS, Jacobs DR, Montoye HJ, Sallis JF, Paffenbarger RS (1993). Compendium of physical activities: classification of energy costs of human physical activities. Med Sci Sports Exerc.

[18] Hegde N, Bries M, Swibas T, Melanson E, Sazonov E (2017). Automatic recognition of activities of daily living utilizing insole-based and wrist-worn wearable sensors. IEEE J Biomed Health Inform.

[19] Pham MH, Elshehabi M, Haertner L, Del Din S, Srulijes K, Heger T (2017). Validation of a step detection algorithm during straight walking and turning in patients with Parkinson’s disease and older adults using an inertial measurement unit at the lower back. Front Neurol.

[20] Warmerdam E, Romijnders R, Geritz J, Elshehabi M, Maetzler C, Otto JC (2021). Proposed mobility assessments with simultaneous full-body inertial measurement units and optical motion capture in healthy adults and neurological patients for future validation studies: study protocol. Sensors.

[21] Hickey A, Del Din S, Rochester L, Godfrey A (2017). Detecting free-living steps and walking bouts: validating an algorithm for macro gait analysis. Physiol Meas.

[22] Storm FA, Buckley CJ, Mazzà C (2016). Gait event detection in laboratory and real life settings: accuracy of ankle and waist sensor based methods. Gait Posture.

[23] Paraschiv-Ionescu A, Newman C, Carcreff L, Gerber CN, Armand S, Aminian K (2019). Locomotion and cadence detection using a single trunk-fixed accelerometer: validity for children with cerebral palsy in daily life-like conditions. J Neuroeng Rehabil.

[24] Bertuletti S, Della Croce U, Cereatti A (2019). A wearable solution for accurate step detection based on the direct measurement of the inter-foot distance. J Biomech.

[25] Kluge F, Del Din S, Cereatti A, Gaßner H, Hansen C, Helbostad JL (2021). Consensus based framework for digital mobility monitoring. PLoS ONE.

[26] Mobilise-D website: www.mobilise-d.eu/

[27] Mazzà C, Alcock L, Aminian K, Becker C, Bertuletti S, Bonci T (2021). Technical validation of real-world monitoring of gait: a multicentric observational study. BMJ Open.

[28] Hollman JH, McDade EM, Petersen RC (2011). Normative spatiotemporal gait parameters in older adults. Gait Posture.

[29] Bonci T, Salis F, Scott K, Alcock L, Becker C, Bertuletti S (2022). An algorithm for accurate marker-based gait event detection in healthy and pathological populations during complex motor tasks. Front Bioeng Biotechnol..

[30] Salis F, Bertuletti S, Bonci T, Della Croce U, Mazzà C, Cereatti A (2021). A method for gait events detection based on low spatial resolution pressure insoles data. J Biomech.

[31] Salis F, Bertuletti S, Scott K, Caruso M, Bonci T, Buckley E, et al. A wearable multi-sensor system for real world gait analysis. In 2021 43rd Annual International Conference of the IEEE Engineering in Medicine & Biology Society (EMBC) (pp. 7020–7023). IEEE; 2021. 10.1109/EMBC46164.2021.9630392 10.1109/EMBC46164.2021.9630392 34892719

[32] Smith E, Cusack T, Blake C (2016). The effect of a dual task on gait speed in community dwelling older adults: a systematic review and meta-analysis. Gait Posture.

[33] Kowalsky DB, Rebula JR, Ojeda LV, Adamczyk PG, Kuo AD (2021). Human walking in the real world: interactions between terrain type, gait parameters, and energy expenditure. PLoS ONE.

[34] Shah VV, McNames J, Mancini M, Carlson-Kuhta P, Spain RI, Nutt JG (2020). Laboratory versus daily life gait characteristics in patients with multiple sclerosis, Parkinson’s disease, and matched controls. J Neuroeng Rehabil.

[35] Warmerdam E, Hausdorff JM, Atrsaei A, Zhou Y, Mirelman A, Aminian K (2020). Long-term unsupervised mobility assessment in movement disorders. Lancet Neurol.

