# Supplementary material for: Design and validation of a multi-task, multi-context protocol for real-world gait simulation
Source: J Neuroeng Rehabil. 2022 Dec 16;19:141. doi: 10.1186/s12984-022-01116-1 (PMC9754996; doi:10.1186/s12984-022-01116-1)
Supplement: Supplementary file 1 — Additional file 1: Table S1. Inclusion and exclusion criteria adopted for the different disease cohorts. [file 12984_2022_1116_MOESM1_ESM.docx]

**Table S1: Inclusion and exclusion criteria adopted for the different disease cohorts**

| **Group** | **Inclusion criteria** | **Exclusion criteria** |
| --- | --- | --- |
| All groups | -able to walk 4 meters independently with or without walking aids  -able to give informed consent  -willingness to wear the sensor set-ups during the study  -shoe size 36 (3 UK) or above  -able to read and write in first language of the respective country  -Montreal Cognitive Assessment (MoCA) >15  -available for home /office visit during study period | -occurrence of any of the following 3 months prior to inclusion: myocardial infarction, hospitalization for unstable angina, stroke, coronary artery bypass graft (CABG), percutaneous coronary intervention (PCI), implantation of a cardiac resynchronization therapy device (CRTD)  -current medical condition that could interfere with the patient’s compliance |
| OHA | 65+ years of age |  |
| PD | -aged 18+ years  -Diagnosis of PD according to the Movement Disorders Society criteria | -impaired mobility related to non-PD causes, as judged by the investigator |
| MS | -aged 18+ years  -Diagnosis of MS based on the revised McDonald’s criteria | -impaired mobility related to non-MS causes, as judged by the investigator |
| PFF | -65+ years of age  -surgical treatment (fixation or arthroplasty) for a low-energy fracture of the proximal femur (ICD-10 diagnosis S72.0, S72.1, S72.2) as diagnosed on X-rays of the hip and pelvis within last 12 months | -impaired mobility related to non-PFF causes, as judged by the investigator |
| COPD | -≥45 years of age  -Diagnosis of COPD (post-bronchodilator forced expiratory volume in the first second (FEV_1_) to forced vital capacity (FVC) ratio <0.70)  -clinical stability, defined as at least 4 weeks without antibiotics and/or oral corticosteroids to treat either a moderate or severe exacerbation  -non-smokers, current or ex-smokers with a smoking history equivalent to at least 10 pack years (1 pack year = 20 cigarettes smoked per day for 1 year) | -having undergone major lung surgery (e.g. lung volume reduction, lung transplant)  -having a lung tumor  -primary respiratory diseases other than COPD (e.g. asthma)  - impaired mobility related to non-COPD causes, as judged by the investigator |
| CHF | -≥45 years of age  - Diagnosis of chronic heart failure with a grading of II–IV of the New York Heart Association Classification | - history of COPD ≥GOLD III  - impaired mobility related to non-CHF causes, as judged by the investigator |
