# Supplementary material for: Design and validation of a multi-task, multi-context protocol for real-world gait simulation
Source: J Neuroeng Rehabil. 2022 Dec 16;19:141. doi: 10.1186/s12984-022-01116-1 (PMC9754996; doi:10.1186/s12984-022-01116-1)
Supplement: Supplementary file 3 — Additional file 3: Table S2. Descriptive statistics of the walking speed captured in the laboratory-based assessment and 2.5 hour of unsupervised monitoring in the participants habitual environment for each cohort. [file 12984_2022_1116_MOESM3_ESM.docx]

**Table S2: Descriptive statistics of the walking speed captured in the laboratory-based assessment and 2.5 hour of unsupervised monitoring in the participants habitual environment for each cohort.**

|  | Walking Speed (m/s) | | | | | | | | | |
| --- | --- | --- | --- | --- | --- | --- | --- | --- | --- | --- |
| Cohort | Laboratory-based assessment | | | | | 2.5-hour unsupervised monitoring | | | | |
|  | Mean | STD | 25-perc | Median | 75-perc | Mean | STD | 25-perc | Median | 75-perc |
| OHA (n=20) | 0.83 | 0.30 | 0.61 | 0.85 | 1.04 | 0.63 | 0.29 | 0.42 | 0.56 | 0.79 |
| PD (n=19) | 0.70 | 0.30 | 0.50 | 0.66 | 0.86 | 0.66 | 0.34 | 0.39 | 0.61 | 0.85 |
| MS (n=19) | 0.77 | 0.30 | 0.54 | 0.73 | 0.98 | 0.70 | 0.32 | 0.46 | 0.64 | 0.87 |
| PFF (n=16) | 0.60 | 0.33 | 0.37 | 0.52 | 0.80 | 0.50 | 0.21 | 0.35 | 0.48 | 0.63 |
| COPD (n=17) | 0.80 | 0.31 | 0.59 | 0.82 | 1.01 | 0.61 | 0.22 | 0.46 | 0.59 | 0.74 |
| CHF (n=11) | 0.78 | 0.33 | 0.52 | 0.71 | 1.00 | 0.79 | 0.34 | 0.53 | 0.77 | 1.06 |
